# Editorial: An approach of brain derived extracellular vesicles in diagnosis and prognosis of brain pathologies

**DOI:** 10.3389/fnint.2022.1025277

**Published:** 2022-10-13

**Authors:** Shahnaz Qadri, Aijaz S. Parray, Ikhlak Ahmed

**Affiliations:** ^1^Irma Lerma Rangel College of Pharmacy, Texas A&M University, Kingsville, TX, United States; ^2^Neuroscience Institute, Hamad Medical Corporation, Doha, Qatar; ^3^Department of Human Genetics, Sidra Medicine, Ar-Rayyan, Qatar

**Keywords:** neuronal exosomes, neurodegenerative disease, CNS biomarkers, extracellular vesicles (EVs), stroke

Extracellular vesicles famous by the name of exosomes are potential diagnostic and therapeutic markers for almost all types of diseases. However, very little is known about exosomes derived from brain tissue because of minimal access to the inner cranial tissues, selective permeability of blood-brain barrier, and delicacy of neuronal tissues. Nevertheless, development of research on neuronal exosomes is significantly rising. Long et al. studies, determined the scientometric research on exosome in neuroscience between 2005 and 2021. Analysis included the number of research articles published, citation bursts, and journal impact. Overall, the scientometric analysis was performed from 856 articles which suggested a significant increase of exosome research in neuroscience using several tools like Carrot2 online system, a clustering and visualizing platform to identify Research Topics about exosomes in neuroscience. These efforts reveal that Alzheimer's disease and mesenchymal stem cells are the major research interests of exosome research in neuroscience and suggest that exosome-based therapeutic technology might be state-of-the-art in the foreseeable future.

It is still challenging to separate neuronal exosomes from plasma or serum blood to develop diagnostic markers for many known neurodegenerative diseases. The research article by Moussa et al. focused on characterizing neuronal exosomes from blood plasma, suggesting NCAM-positive EVs may be suitable neuronal markers in exosomes. This study utilizes brain organoids and stem cells to demonstrate NCAM as a neuronal biomarker in exosomes.

Brain comprises both neuronal and non-neuronal tissues; this Research Topic focuses on the function of brain-derived extracellular vesicles (EVs) in the central nervous system (CNS) and their role in the diagnosis and prognosis of brain pathologies. Gassama and Favereaux summarized EVs' physiology, pathology, and therapeutic perspectives in the CNS. They outlined the role of EVs released from Neurons and non-neuronal cells (Astrocytes and Glial cells) as our primary source for inter-cellular communication. In addition to the physiological function, Huo et al. added the value of intercellular communication by the neuronal-derived exosomes. Neuronal EVs are shown to carry chemokines, transfer secondary messenger molecules to the cells, and contain ion channels such as AMPA receptors and Synaptotagmin4. This transfer of functional proteins and ion channels changes the post-synaptic plasticity in the recipient cells. The EVs released from neurites during depolarization is enriched with miR-132, known to regulate the Notch signaling pathway. EVs released from Astrocytes show the maturation of oligodendrocytes, neurite growth, neurite protection during oxidative stress and possess unique surface markers.

On the contrary, Gassama and Favereaux shed light on pathological implications of EVs containing miRNA and pathology-related proteins such as amyloid proteins, alpha-synuclein, or tau-protein that can propagate in surrounding tissues or initiate an undesired epigenetic regulation in recipient cells. Again, contrary to the adverse effects of EVs, EVs could be the leading source for diagnostic markers of CNS pathology such as Glioblastoma, Alzheimer's disease, Huntington's disease, and Multiple sclerosis. In addition, Gassama and Favereaux summarized the therapeutics of EVs to deliver drugs across the blood-brain barrier such as siRNA and Curcumin. Huo et al. added the valued information on the effect of external stimuli causing neurons' excessive secretion of exosomes. These influences are mainly from the depolarization of neurons. Exosomes derived from Microglia exposed to LPS showed exosomes containing IL-4, extracellular ATP activating P2X7 receptors initiate secretion of exosomes from microglial cells.

Similarly, astrocytes released exosomes showed specific markers like glutamine aspartate transporter (GLAST) and glutamine synthetase (GLUL). Furthermore, studies show free ATP bind to P2X7R induces IL-1β in astrocyte-derived exosomes, and exposure of exosome to neurons initiate the differentiation of dendrites and synapses. In addition, Huo et al. in their review paper, focused on the exposure of cytotoxic proteins to astrocytes releasing exosomes rich in PAR-4 that promote apoptosis, and also discussed the function of amyloid, ceramide, and prions in the regulation of exosome secretion which has a crucial role in neuroprotection. Also, Huo et al. discussed the role of exosomes in three major neurodegenerative diseases: Alzheimer's, Parkinson's, and Amyotrophic Lateral Sclerosis. Stroke is a cerebrovascular accident or transient ischemic attack in which blood flow to brain is blocked leading to immediate death or paralysis. As such, there are no clinically approved biomarkers to identify early symptoms of a stroke. The review of Xu et al. focused on exosomal miRNA studies in stroke patients. They summarized the outcome of clinical studies for developing the diagnostic biomarker of ischemic stroke. This study also covers the role of miRNA in the pathogenesis of stroke by cerebral Ischemia. Mainly, animal and *in vitro* studies are employed to understand miRNA's effect and underlying mechanism in a neuro-protective role, such as promoting angiogenesis, protecting from oxidative stress, and inhibiting apoptosis.

Preterm babies born are at high risk to brain injuries causing permanent motor disabilities. The ability of exosomes to cross the blood-brain barrier likewise crosses the placental barrier. A comprehensive review by Gamage and Fraser discusses causes of prenatal injury, the injury outcome, mainly cerebral palsy, and the possible treatment with extracellular vesicles isolated from mesenchymal stem cells. Only a limited number of studies are employed using animal models, which shows EVs have a significant therapeutic effect in repairing damage associated with prenatal brain injury. This review also discussed the uptake of exosomes or EVs by neurons, glial cells, and their downstream signaling effects. Furthermore, Gamage and Fraser discussed the circulation of exosomes derived from fetal CNS into maternal blood; a potential biomarker for early prenatal brain damage.

Overall neuronal exosome inquiries reveal the birth of a new field in neuroscience that will contribute to a better understanding of neuronal communication that may ultimately provide more comprehensive options for early diagnosis and better therapeutics.

## Author contributions

SQ wrote the manuscript. AP and IA revised the manuscript and gave input. All authors contributed to the article and approved the submitted version.

## Conflict of interest

Author AP was employed by Hamad Medical Corporation. The remaining authors declare that the research was conducted in the absence of any commercial or financial relationships that could be construed as a potential conflict of interest.

## Publisher's note

All claims expressed in this article are solely those of the authors and do not necessarily represent those of their affiliated organizations, or those of the publisher, the editors and the reviewers. Any product that may be evaluated in this article, or claim that may be made by its manufacturer, is not guaranteed or endorsed by the publisher.

